# Midline Shift Induced by the Drainage of Cerebrospinal Fluid in Three Patients With External Decompression

**DOI:** 10.7759/cureus.44355

**Published:** 2023-08-29

**Authors:** Shoko M Yamada, Naotaka Iwamoto, Yusuke Tomita, Ririko Takeda, Makoto Nakane

**Affiliations:** 1 Neurosurgery, Teikyo University Mizonokuchi Hospital, Kawasaki, JPN

**Keywords:** decompressive craniectomy, lumbar drainage, kernohan notch, atmospheric pressure, paradoxical herniation

## Abstract

It is not rare that progressive hydrocephalus worsens clinical conditions in a patient with external decompression and drainage or shunt surgery is required. However, spinal drainage or shunt surgeries potentially carry a risk of causing paradoxical herniation in a patient with decompressive craniectomy, particularly in a comatose case with wide craniectomy. Careful and strict observations are necessary for such patients. In our three comatose cases with craniectomy, paradoxical herniation occurred due to excessive drainage after 5-7 days of shunt surgery and lumbar drainage, although the drainage pressure was set at more than 10 cmH_2_O. Fortunately, in the three cases, the herniation improved within a few days after the drain was clamped and the bed was flattened. However, the Trendelenburg position and epidural blood patch might be necessary if paradoxical herniation occurs acutely after lumbar puncture or drainage because delayed resolution can be fatal in the herniation.

## Introduction

Cerebral herniation is defined as a shift of cerebral tissue from its normal location into an adjacent space as a result of focal brain edema or the strong focal compression of normal brain tissue [1]. In most cases, herniation is associated with increased intracranial pressure (ICP) [1,2]. However, cerebral herniation can be induced by decreased ICP caused by lumbar drainage or shunt surgery in patients with large decompressive hemicraniectomy; this is called paradoxical herniation [3-10]. In this paper, the authors present three cases of paradoxical herniation and discuss early detection and management.

## Case presentation

Case 1

**Figure 1:**
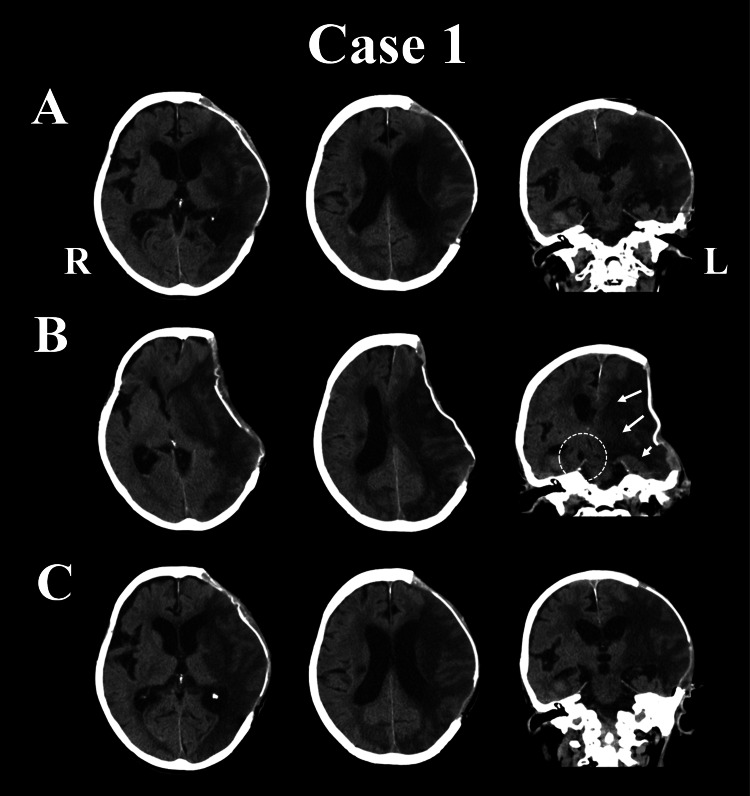
CT findings in Case 1. (A) Mild ventriculomegaly is apparent. The left hemisphere is protruding from the cranium, and the skin at the site of the bone defect is bulging. (B) There is a remarkable sinking of the skin at the site of the bone defect, causing a midline shift to the side contralateral to the bone defect. Because the bone defect has been compressed inward and downward by atmospheric pressure (white arrows), the midbrain has shifted to the right side, resulting in the Kernohan notch phenomenon (white circle). (C) The ventricle has enlarged, the midline shift has resolved, and the compression of the midbrain by the cerebellar tent is less marked. CT: computed tomography

A 48-year-old female underwent decompressive hemicraniectomy for an extensive cerebral infarction caused by the occlusion of the left middle cerebral artery due to cardiogenic embolism. Before surgery, her consciousness level was 5 on the Glasgow Coma Scale (GCS) (eye opening, 1; verbal response, 1; and motor response, 3) but recovered to GCS 11 (E4V2M5) two months after the surgery. Before a cranioplasty, lumbo-peritoneal shunt was preceded because a computed tomography (CT) scan three months after the external decompression showed that her ventriculomegaly had progressed and the bone defect was still bulging. Shunt pressure was controlled by a 1.5 (100 mmH_2_O) Strata NSC valve (Medtronic plc, Minneapolis, MN). Five days later, the patient began vomiting and would not open her eyes without strong stimulation (GCS 6; E2V1M3). The patient demonstrated anisocoria with a left pupillary diameter of 4.0 mm and a right pupillary diameter of 2.5 mm, and the light reflex in the right pupil was sluggish. Judging from the appearance, there was only mild skin depression, and the skin tension was soft. CT demonstrated apparent sinking of the skin at the site of the bone defect with a midline shift to the side contralateral to the bone defect. A coronal view showed the obvious compression of the brainstem by the cerebellar tent, this being caused by inward and downward compression due to atmospheric pressure. Increasing the shunt pressure to 2.5 (210 mmH_2_O) resulted in the gradual bulging of the skin at the site of the bone defect accompanied by the patient regaining the ability to open her eyes normally (GCS 11; E4V2M5) within five days. CT showed ventriculomegaly and the resolution of the midline shift (Figure 1). Afterward, by controlling the Strata NSC valve pressure to 1.5 (100 mmH_2_O) for three days prior to surgery, the patient successfully underwent cranioplasty two weeks after the shunt surgery.

Case 2

**Figure 2:**
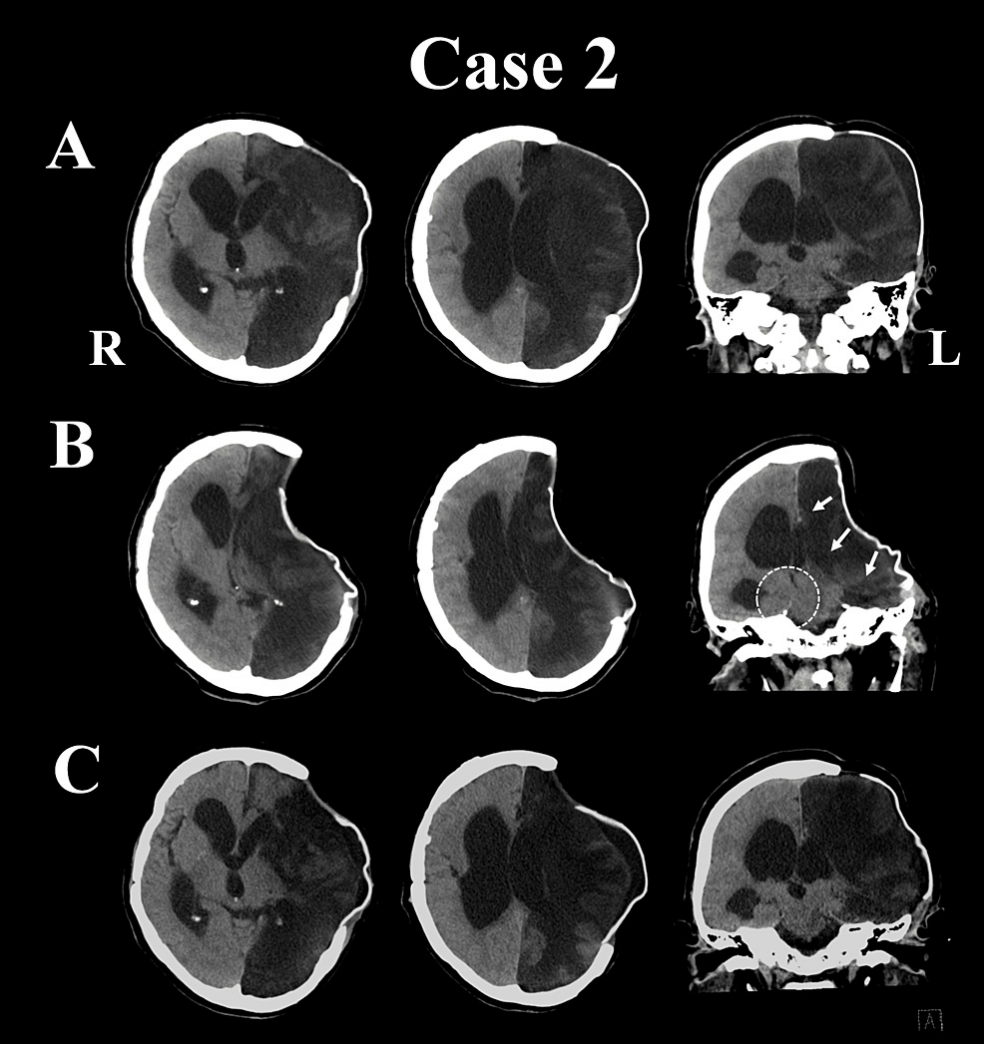
CT findings in Case 2 (A) There is a significant enlargement of the lateral ventricles, especially on the right. The skin at the site of the decompressive craniectomy is bulging as a result of the diffuse edema of the left hemisphere. (B) The skin at the site of the bone defect has sunk considerably. The midline shift contralateral to the bone defect seems mild; however, because of the inward and downward pressure at the site of the bone defect (white arrows), the right side of the midbrain is compressed by the cerebellar tent (white circle). (C) The size of the right ventricle is mildly decreased and that of the left ventricle is enlarged. There is improvement in the midline shift and compression of the midbrain by the cerebellar tent. CT: computed tomography

A 72-year-old male underwent external decompression for an extensive cerebral infarction caused by the occlusion of the left internal carotid artery due to arteriosclerosis. He was comatose before surgery (GCS 5; E1V1M3) but was able to open his eyes spontaneously and move his left limbs with some painful stimuli (GCS 9; E4V1M4) one month after surgery. The bone defect was still bulging 1.5 months after hemicraniectomy, the activity of the patient gradually slowed down opening his eyes only by painful stimulation, and the patient's ability in excreting sputa deteriorated resulting in mild aspiration pneumonia. CT displayed remarkable ventriculomegaly; then, lumbar drainage was performed, and the pressure was controlled at 12 cmH_2_O above the external auditory meatus level. In a few days after the drainage, the patient could open his eyes spontaneously. But five days after the drainage, the patient would not open his eyes and had poor limb movement by painful stimulation (GCS 5; E1V1M3). The diameter of the left pupil was 4 mm with sluggish light reflex, while the right pupil was 2.0 mm with prompt reflex. Externally, the skin at the bone defect was mildly sunken, and the skin was soft without identifying high tension. CT showed a midline shift to the side contralateral to the bone defect with the sinking of the skin, and the right ventriculomegaly was still present. A coronal view showed the compression of the midbrain by the cerebellar tent because of the inward and downward pressure from the bone defect. After clamping the drain for three days, the patient regained the ability to open his eyes normally (GCS 9; E4V1M4). CT showed improvement in the midline shift, and the drainage was removed (Figure 2).

Case 3

**Figure 3:**
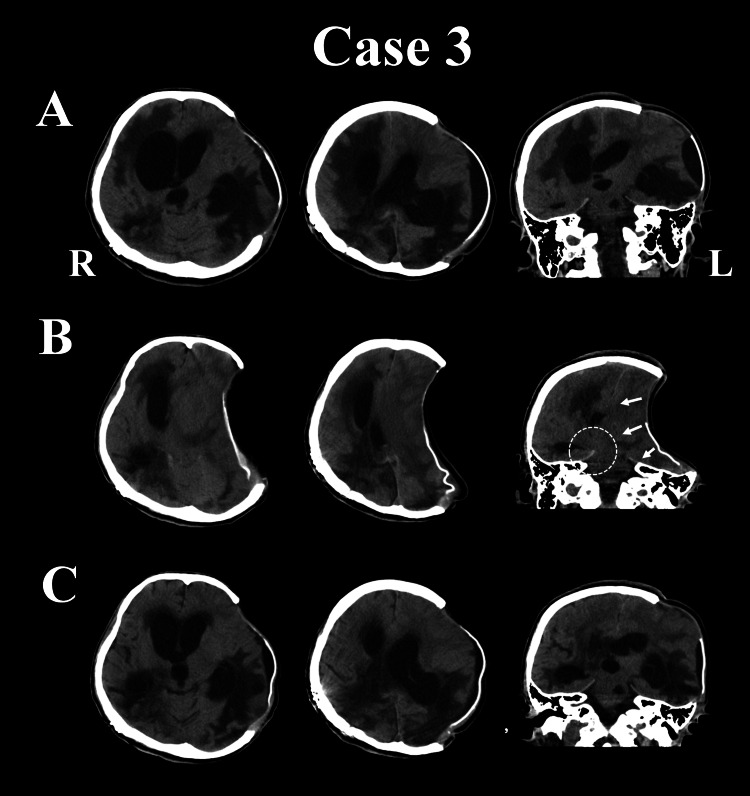
CT findings in Case 3 (A) The lateral ventricles are tremendously enlarged, especially on the right side, with obvious periventricular lucency. Additionally, the skin at the site of the decompressive craniectomy is bulging, forming a thin subdural hygroma. (B) There is severe skin sinking at the site of the bone defect, and the left ventricle is difficult to recognize because of the compression. The midline has shifted significantly to the side contralateral to the bone defect, suggesting impending brain herniation with the Kernohan notch phenomenon (white circle) caused by the inward and downward compression (white arrows). (C) The midline shift has improved, and the periventricular lucency at the right anterior horn has resolved. The sulci and ambient cistern have become clear, and the pressure on the midbrain has also eased. CT: computed tomography

A 33-year-old male demonstrated left hemiplegia due to multiple infarctions including the brainstem, cerebellum, and bilateral hemispheres, but he was almost alert at admission. Heparin was administered on admission, but the treatment was quit soon after reaching to the diagnosis of infectious endocarditis, and the administration of antibiotics was initiated. The consciousness level of the patient deteriorated suddenly in the next morning (GCS 5; E1V1M3), due to massive hemorrhage at the left temporoparietal lobe identified on CT. The patient underwent hematoma removal and left external decompression. Four days later, another hemorrhage occurred in the right occipitoparietal lobe with intraventricular hemorrhage, necessitating the removal of hematoma by craniotomy. In one month, he was able to open his eyes spontaneously and keep his eyes on someone, but no nodding movements or utterances were heard from him (GCS 8; E4V1M3). As the skin at the craniectomy site swelled, the patient did not open his eyes spontaneously. Two months after the surgery, excessive enlarged ventricles with definite periventricular lucency on CT prompted the implementation of lumbar drainage, with pressure being controlled at 15 cmH_2_0 above the level of the external auditory meatus. He recovered to open his eyes spontaneously within a few days after the drainage. But the skin at the site of external decompression gradually became depressed, and he began to vomit and lost consciousness (GCS 4; E1V1M2) in one week after the drainage. His left pupil was 5 mm in diameter with absent light reflex, whereas his right pupil was 2.5 mm in diameter with prompt light reflex. Moderate skin sinking at the bone defect was identified, but the skin was still soft, and wave motion occurred by touching. A head CT showed a marked midline shift with severe sinking of the skin at the site of the bone defect. A coronal view clearly showed that the midbrain was compressed by the cerebellar tent and that this was induced by the inward and downward pressure at site of the bone defect. After the drain had been clamped for three days, the size of his pupils equalized, and he was able to open his eyes (GCS 7; E3V1M3). CT showed the resolution of the midline shift, and the drainage was removed (Figure 3).

## Discussion

Even in cases with decompressive craniectomy, lumbar drainage or shunt surgeries may be necessary if the ICP increases with high tension at a bone defect scalp due to the progression of hydrocephalus, causing deteriorated activities and consciousness level. The Monro-Kellie hypothesis [11] is not indicated in patients undergoing decompressive craniectomy, and the intracranial volume is not maintained constant. Therefore, in a patient with decompressive craniectomy, lumbar drainage or shunt surgery carries a risk to cause sinking skin flap syndrome (SSFS) or trephined syndrome, progressing to paradoxical herniation [9,10,12-15]. The main cause of the paradoxical herniation is an excessive cerebrospinal fluid (CSF) reduction due to inadequate drainage management [4-6]. Even if the drainage or shunt pressure is controlled at a higher than normal level, over-drainage could occur because the bone defect portion was constantly compressed by atmospheric pressure (1013 hPa=1033 cmH_2_O) [5,6]. Lumbar drainage and shunt pressure were set at 10-15 cmH_2_0 in our cases, but paradoxical herniation occurred. The authors consider the following: 1) In the case with shunts, the management should be initiated with the highest shunt pressure setting and gradually lowered checking the scalp tension at the bone defect, and 2) in the case of lumbar drainage, the drainage must be controlled by excreted CSF volume rather than by pressure, determining the amount of drained CSF for a certain time, and the drainage is intermittently clamped when the planned amount is drained and then released again. Zhao et al. reported nine cases of SSFS, which was managed by a continuous drainage from a lumbar catheter with a volume of 200-300 mL/day [10]. Then, it might be better to keep the drainage volume below 200 mL/day for the prevention of paradoxical herniation.

Several cases of paradoxical herniation have been reported in patients necessitating decompressive craniectomy due to cerebral hemorrhage, subarachnoid hemorrhage, ischemic stroke, and head trauma [3-10], but no association between diseases and the frequency of paradoxical herniation has been stated. According to Sarov et al., there was no statistical association of age, gender, and the size of brain injury to the incidence of paradoxical herniation; however, the wideness of the decompressive craniectomy area and the daily amount of CSF drainage were significantly related to SSFS and paradoxical herniation [6]. In SSFS, clinical symptoms such as motor weakness, cognitive deficits, language deficits, headache, seizures, and lethargy are commonly present suggesting over-drainage in patients [5,15]. However, in patients with impaired consciousness, these early symptoms may hardly be recognized. In paradoxical herniation, the brain is compressed from the parietotemporal to the central region of the brain at the bone defect site; the midbrain is the most affected area; therefore, anisocoria may be the first sign to be identified, and the Kernohan notch phenomenon might be identified [16,17].

In our cases, paradoxical herniation was identified in 5-7 days after performing lumbar drainage or shunt surgery. But the duration of the herniation after lumbar puncture, lumbar drainage, or shunt surgeries varies between reports; for example, paradoxical herniation occurred after 1-2 hours or six weeks after lumbar puncture [3,8,18,19], 24 hours after lumbar drainage [9], and 2-3 months after shunt surgery [9]. The principles of treatment for paradoxical herniation are to stop the drainage of CSF (clamp the drain), to return CSF to the ventricles (the Trendelenburg position), and to accelerate the production of CSF (intravenous fluid infusion). The immediate removal of the lumbar drain is not recommended as CSF may continue to leak into the epidural space after the removal. One case was reported to be dead, reaching to irreversible paradoxical herniation within a few hours after lumbar puncture even though the patient was in the Trendelenburg position immediately [3]. If paradoxical herniation occurs after the removal of lumbar puncture or lumbar drain, emergent lumbar epidural blood patch placement might be necessary [20].

## Conclusions

Paradoxical herniation is sometimes irreversible and results in death. Early detection is crucial to prevent paradoxical herniation in patients with disturbed consciousness. As the herniation is characterized by the compression of the midbrain, it is important to observe the pupils in such patients, and if a left-right difference in pupil size develops, the progression of the herniation should be suspected. The first remedy includes stopping drainage, placing the patients in the Trendelenburg position, and giving a sufficient amount of intravenous fluid infusion; however, if these treatments are ineffective, emergent epidural blood patch placement should be performed without hesitation.
